# Reassessment of the Four Yield-related Genes *Gn1a*, *DEP1*, *GS3*, and *IPA1* in Rice Using a CRISPR/Cas9 System

**DOI:** 10.3389/fpls.2016.00377

**Published:** 2016-03-30

**Authors:** Meiru Li, Xiaoxia Li, Zejiao Zhou, Pingzhi Wu, Maichun Fang, Xiaoping Pan, Qiupeng Lin, Wanbin Luo, Guojiang Wu, Hongqing Li

**Affiliations:** ^1^Key Laboratory of Plant Resources Conservation and Sustainable Utilization, South China Botanical Garden, Chinese Academy of SciencesGuangzhou, China; ^2^Guangdong Provincial Key Laboratory of Applied Botany, South China Botanical Garden, Chinese Academy of SciencesGuangzhou, China; ^3^Guangdong Provincial Key Lab of Biotechnology for Plant Development, South China Normal UniversityGuangzhou, China

**Keywords:** CRISPR/Cas9 system, gene editing, *Oryza sativa* L., yield-related genes, yield-related traits

## Abstract

Clustered Regularly Interspaced Short Palindromic Repeats (CRISPR)-associated (Cas) systems have been successfully used as efficient tools for genome editing in a variety of species. We used the CRISPR/Cas9 system to mutate the *Gn1a* (Os01g0197700), *DEP1* (Os09g0441900), *GS3* (Os03g0407400), and *IPA1* (Os08g0509600) genes of rice cultivar Zhonghua 11, genes which have been reported to function as regulators of grain number, panicle architecture, grain size and plant architecture, respectively. Analysis of the phenotypes and frequencies of edited genes in the first generation of transformed plants (T0) showed that the CRISPR/Cas9 system was highly efficient in inducing targeted gene editing, with the desired genes being edited in 42.5% (*Gn1a*), 67.5% (*DEP1*), 57.5% (*GS3*), and 27.5% (*IPA1*) of the transformed plants. The T2 generation of the *gn1a*, *dep1*, and *gs3* mutants featured enhanced grain number, dense erect panicles, and larger grain size, respectively. Furthermore, semi-dwarf, and grain with long awn, phenotypes were observed in *dep1* and *gs3* mutants, respectively. The *ipa1* mutants showed two contrasting phenotypes, having either fewer tillers or more tillers, depending on the changes induced in the OsmiR156 target region. In addition, we found that mutants with deletions occurred more frequently than previous reports had indicated and that off-targeting had taken place in highly similar target sequences. These results proved that multiple regulators of important traits can be modified in a single cultivar by CRISPR/Cas9, and thus facilitate the dissection of complex gene regulatory networks in the same genomic background and the stacking of important traits in cultivated varieties.

## Introduction

Rice (*Oryza sativa* L.) is the most important food crop in the world, feeding over half of the global population. In modern rice farming, high yield has accordingly become one of the major objectives of breeders and growers over recent decades (Wang and Li, 2008; Xing and Zhang, 2010). Rice yield per plant is determined by three component traits: number of panicles per plant, number of grains per panicle, and grain weight (Wang and Li, 2008; Xing and Zhang, 2010). To date, a number of genes have been shown to influence these yield traits: *MONOCULM 1* (*MOC 1*), *TEOSINTE BRANCHED1* (*OsTB1*), and *IDEAL PLANT ARCHITECTURE1* (*IPA1*) control tillering in rice (Li et al., 2003; Jiao et al., 2010; Minakuchi et al., 2010; Miura et al., 2010); *GRAIN NUMBER 1a* (*Gn1a*), *DROUGHT AND SALT TOLERANCE* (*DST*), *IPA1*, and *Grains.Height.Date-7* (*Ghd7*) regulate grain number (Ashikari et al., 2005; Xue et al., 2008; Jiao et al., 2010; Miura et al., 2010; Li S. et al., 2013);*Grain Size* (*GS3*), *Grain Weight* (GW2, GW5), *Squamosa Promoter Binding Protein-like 16* (*OsSPL16*), *Ser/Thr phosphatase* (*OsPPKL1*), and *GNAT-like Protein* (*OsglHAT1*) regulate grain size (Fan et al., 2006; Song et al., 2007; Shomura et al., 2008; Weng et al., 2008; Mao et al., 2010; Wang et al., 2012; Zhang et al., 2012; Song et al., 2015; Wang S. et al., 2015) and *DENSE AND ERECT PANICLE* (*DEP1*) controls panicle size (Huang et al., 2009). The *Gn1a* allele in Habataki (an indica cultivar) harbors a mutation in the OsCKX2 gene, which encodes a CK oxidase/dehydrogenase (CKX) that catalyzes the degradation of active cytokinin. Mutation or reduced expression of *Gn1a* causes accumulation of CK in inflorescence meristems and increases the number of reproductive organs, resulting in enhanced grain production (Ashikari et al., 2005). Plants with *IPA1* containing a mutation in the miR156 cleavage site, in Taichung Native 1 (an indica cultivar) and Aikawa and Shaoniejing (japonica cultivars), displayed the ideal plant architecture (IPA), which includes low tiller numbers, few unproductive tillers, more grains per panicle, and thick and sturdy stems, substantially enhancing rice grain yield (Jiao et al., 2010; Miura et al., 2010). The mutant *DEP1* allele in Shennong 265 and Jiahua 1 (japonica cultivars) profoundly changes rice inflorescence architecture, resulting in a dense and erect panicle, and a consequent increase in grain yield (Huang et al., 2009). *GS3*, a gene which colocates with a major quantitative trait locus for grain size, contains four putative domains: a plant-specific organ size regulation (OSR) domain at the N terminus, a transmembrane domain, a tumor necrosis factor receptor/nerve growth factor receptor (TNFR/NGFR) family cysteine-rich domain, and a von Willebrand factor type C (VWFC) at the C terminus. Loss of function of the OSR domain in Minghui 63 (an indica cultivar) results in long grains and a consequent increase in grain weight; while loss of function at the C terminus produces very short grains (Mao et al., 2010). Thus, the *Gn1a*, *IPA1*, *DEP1I*, and *GS3* genes offer potential for manipulating yield-related traits. However, as shown by these examples, yield-related genes have pleiotropic effects on plant development in addition to their effects in regulating yield, and it will therefore be important to carefully assess the functions of the genes in different cultivar backgrounds and choose suitable combinations of alleles for rice breeding. Prior to doing this, it is first necessary to test the effects of mutating the *Gn1a*, *IPA1*, *DEP1*, and *GS3* genes in a single cultivar, and determine whether loss-of-function mutants of these four genes have the high yield phenotypes reported in previous work.

A recently developed CRISPR/Cas9 system employing a Cas9 endonuclease and a guide RNA complex has shown very high efficiency for targeted gene editing in a variety of species (Cong et al., 2013; Feng et al., 2013; Jiang et al., 2013; Mali et al., 2013; Nekrasov et al., 2013; Shan et al., 2013). The first reports of CRISPR/Cas9 editing in plants appeared in 2013, describing successful application to *Arabidopsis* (*Arabidopsis thaliana*) (Jiang et al., 2013; Li J.F. et al., 2013), tobacco (*Nicotiana benthamiana*) (Jiang et al., 2013; Nekrasov et al., 2013), sorghum (*Sorghum bicolor*), and wheat (*Triticum aestivum*) (Jiang et al., 2013; Shan et al., 2013); there have also been reports for rice (Jiang et al., 2013; Shan et al., 2013; Zhang et al., 2014; Zhou et al., 2014; Ma et al., 2015; Xu et al., 2015), sweet orange *(Citrus sinensis*) (Jia and Wang, 2014), maize (*Zea mays*) (Liang et al., 2014), Chinese white poplar (*Populus tomentosa* Carr.) (Fan et al., 2015), soybean (*Glycine max*) (Jacobs et al., 2015), and tomato (*Solanum lycopersicum* L.) (Brooks et al., 2014). In rice, it was possible to obtain a high frequency of homozygous or bi-allelic mutations in T0 plants, and modifications to genes in T0 plants were shown to persist into the next generation without the occurrence of any detectable new mutations or reversions (Zhang et al., 2014). Thus, CRISPR/Cas9 technology makes it possible to rapidly and precisely edit specific plant genes of interest to achieve the desired outcomes.

Here we report the application of CRISPR/Cas9 technology (Ma et al., 2015) to specifically induce mutagenesis of the *Gn1a*, *DEP1*, *GS3*, and *IPA1* genes (**Table 1**) in the rice cultivar Zhonghua 11 (a japonica cultivar). We found that plants mutated in the above four genes by CRISPR/Cas9 showed similar phenotypes to those described in some previous reports. Thus, editing of the *Gn1a*, *DEP1*, *GS3*, and *IPA1* genes can work in different genomic backgrounds, offering the potential for plant breeding strategies to improve yield traits in the varieties that are currently cultivated.

**Table 1:** Details of the four genes modified in this research.

Gene	RAPDB_Locus	Molecular function	Traits	Reference
*Gn1a*	Os01g0197700	Cytokinin dehydrogenase2	Grain number per panicle	Ashikari et al., 2005
*DEP1*	Os09g0441900	γ subunit of G protein	Plant height Erect panicle Grain size	Huang et al., 2009; Taguchi-Shiobara et al., 2011; Sun et al., 2014
*GS3*	Os03g0407400	γ subunit of G protein	Seed size	Fan et al., 2006; Mao et al., 2010; Botella, 2012
*IPA1*	Os08g0509600	Squamosa promoter binding protein	Plant height and tiller number	Jiao et al., 2010; Miura et al., 2010

## Materials and Methods

### Plant Material and Growth Conditions

Mature seeds were collected from T0 plants, dried, and germinated for 2 days at 37°C in the dark; germinated seeds were then planted in soil and the seedlings were grown under standard greenhouse conditions (16-h light at 30°C/8-h dark at 22°C). The phenotypes of homozygous T2 mutants were investigated.

### Vector Construction

The sgRNA-Cas9 plant expression vectors were kindly provided by Prof. Yaoguang Liu (South China Agriculture University). The vectors were constructed by inserting synthesized oligos into the BsaI site of the vector pYLCRISPR/Cas9(I), which contains a codon-optimized Cas9 driven by a maize ubiquitin promoter, a sgRNA scaffold directed by a rice U6a promoter and the backbone of the binary vector pCAMBIA1300 (CAMBIA, Canberra, Australia) (Supplementary Figure 1) (Ma et al., 2015). The oligos used in constructing the sgRNA vectors for *Gn1a*, *DEP1*, *GS3*, and *IPA1* are listed in Supplementary Figure 1C.

### Rice Transformation

The constructs were introduced into *Agrobacterium tumefaciens* strain EHA105 by electroporation. *Agrobacterium*-mediated transformation of rice (*Oryza sativa* L. ssp. *japonica*. Zhonghua 11) was performed as described (Li and Li, 2003). T0 transgenic plants were used for the detection of mutations.

### Mutation and Off-target Detection

Genomic DNA extraction from leaves of T0 transgenic rice plants (40 independent transgenic lines) was carried out using the sodium dodecyl sulfate (SDS) method (Dellaporta et al., 1983). PCR amplifications were carried out using primer pairs flanking the designated target sites. Mutation detection was performed using CEL I or restriction enzyme assays. For CEL 1 analysis, the PCR products from putative mutants were denatured and annealed, and then subjected to CEL I digestion. Lines with PCR products resistant to CEL1 digestion are homozygous WT or mutants. For restriction enzyme assays, the PCR products were restricted with the appropriate enzyme; mutants will display different bands on an agarose gel compared with the WT. In the cases of *DEP1* and *IPA1* as targets, the PCR products were used for both CEL I and restriction enzyme assays (there is a PvuII site in *DEP1* and a XhoI site in *IPA1* as indicated in **Figures 2** and **4**), and the PCR products of *Gn1a* and *GS3* target region were analyzed using the CEL I assay. Samples in which mutations were detected were cloned into a plasmid vector and 5–6 clones for each sample were sequenced. Off-target events were screened for in the same DNA samples using the CEL I assay. Specific primers (Supplementary Figure 1D) were designed for amplifying fragments possessing high homology with the corresponding sgRNA (**Table 3**).

### Data Collection and Statistical Analysis

For all experiments, three replicates were conducted. Statistical analyses were performed using the SPSS software package version 17.0 (SPSS Inc., Chicago, IL, USA). The significance of differences between controls and treatments was compared at the 0.05 probability level using a one-way analysis of variance least significant difference test.

## Results

### Generation of Gn1a Mutations and Phenotype of the Mutants

**FIGURE 1:**
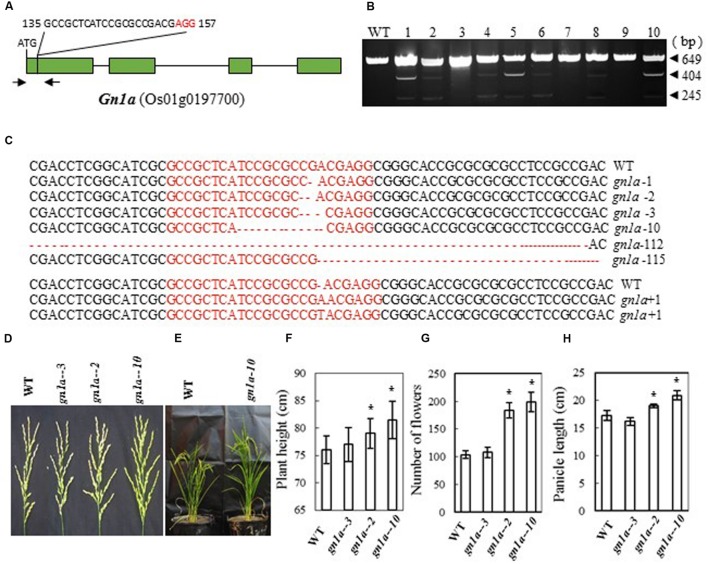
**CRISPR/Cas9-induced *gn1a* mutants and their phenotypes. (A)** Schematic map of the genomic region of *Gn1a* and the sgRNA target site; arrows show the positions of PCR primers used for mutation detection; The PAM motif (NGG) is shown in red; **(B)** Gel electrophoresis of PCR products amplified from the mutated region and digested with CEL I; WT and 1–10 are DNA samples from wild type and different transgenic plants. Arrows show the expected sizes of the bands after CEL I digestion; **(C)** Sequence alignment of the sgRNA target region showing altered bases in different mutant lines; **(D)** Representative pictures showing the morphology of the main panicle; **(E)** Phenotype of the mutant plants grown in the greenhouse; statistics for plant height **(F)**, number of flowers per main panicle **(G)** and panicle length **(H)** in representative mutant plants. Data were collected from 10 to 15 plants per mutant line. * indicates a significant difference in comparison to the WT at *P*<0.05.

Previous studies showed that mutations in *Gn1a* (a 16-bp deletion in the 5′-untranslated region, a 6-bp deletion in the first exon, and three nucleotide changes in the first and fourth exon of the Habataki allele) (Ashikari et al., 2005) result in an increase in the number of grains in the main panicle. We therefore designed a CRISPR/Cas9 construct to target *Gn1a* in the first exon; this was expected to cause mutation in the coding region of the gene (bases 135 to 157 from the ATG codon in the cDNA) and thus inactivate the Gn1a protein (**Figure 1A**). After introducing the construct into rice embryogenic calli by *Agrobacterium*-mediated transformation, many transgenic lines were regenerated. Next, we examined the mutation efficiency by CEL I analysis of PCR products amplified from the target sites, and we observed three bands of 649, 404, and 245 bp (**Figure 1B**), which are the products expected if mutations are present in the target sites. We found 17 mutants in 40 independent transgenic plants, i.e., a mutation rate of 42.5% (**Table 2**). Sequencing of the mutated region revealed that various mutations, including insertion and deletion of different nucleotides, had been produced (**Figure 1C**). The mutant lines were grown in the field and the phenotypes of homozygous T2 mutant plants (with no off-targeting events) were investigated. We found that mutation of the *Gn1a* gene in Zhonghua 11 leading to a frame shift increased plant height, panicle size and number of flowers per panicle (**Figures 1D–H**). The two homozygous mutants *Gn1a-2* (with a 2 bp deletion) and *Gn1a-10* (with a 10 bp deletion) had an average number of flowers per panicle of 184 and 199, respectively, much higher than the number in wild-type plants (104 flowers per panicle) (**Figure 1G**). In addition, plant height and panicle length were significantly increased in the two mutant lines compared with those in wild-type (**Figures 1E,F,H**). As a control, the mutant *Gn1a-3*, which has a 3-base deletion in the coding region, resulting in a single amino acid deletion, showed a similar phenotype to that of wild-type (**Figures 1D–H**). These results suggest that mutation of *Gnla* has similar effects in Zhonghua 11 to those previously reported for this gene in Habataki (Ashikari et al., 2005).

**Table 2:** Percentage of T0 plants found with mutations in the target sequence.

Target gene	No. of plants examined	No. of plants with mutations	Mutation rate (%)	Putative homozygous mutations		Bi-allele mutations	
				Number	%	Number	%
*DEP1*	40	27	67.5	16	40.0	5	12.5
*Gn1a*	40	17	42.5	5	12.5	2	5.0
*GS3*	40	23	57.5	11	27.5	3	7.5
*IPA1*	40	11	27.5	3	7.5	2	5.0
Average	40	19.5	48.8	8.8	21.9	3	7.5

### Generation of DEP1 Mutations and Phenotype of the Mutants

**FIGURE 2:**
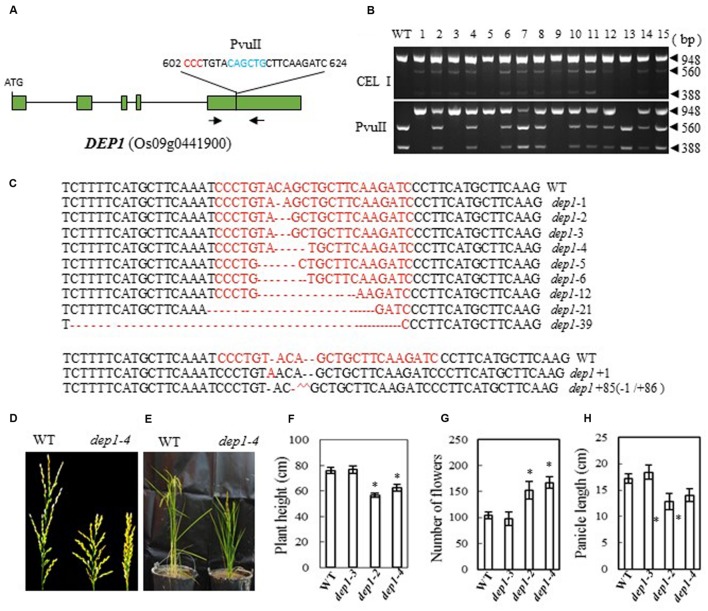
**CRISPR/Cas9-induced *Dep1* mutant plants and phenotype analysis. (A)** Schematic map of the genomic region of *DEP1* and the sgRNA target site; arrows show the positions of PCR primers used for mutation detection; The PAM motif (NGG) is shown in red; Restriction site is shown in blue; **(B)** Gel electrophoresis of PCR products amplified from the mutated region digested with CEL I (upper panel) and PvuII (lower panel); WT and 1–15 are DNA samples from wild type and different transgenic plants. Arrows show the expected band sizes after CEL I or PvuII digestion. **(C)** Sequence alignment of the sgRNA target region showing altered bases in different mutant lines; **(D)** Representative pictures showing the morphology of the main panicle; **(E)** Phenotype of the mutant plants grown in a greenhouse; statistics for plant height **(F)**, number of flowers per main panicle **(G)** and panicle length **(H)** of representative mutant plants. Data were collected from 10 to 15 plants per mutant line. * indicates a significant difference (*P* < 0.05) in comparison to WT controls.

In the Shennong 265 genetic background, plants with a *DEP1* mutation display dense erect panicles; this *DEP1* allele has a 637-bp stretch of the middle of exon 5 replaced by a 12-bp sequence, which has the effect of creating a premature stop codon and consequently a loss of 230 residues from the C terminus of the protein (Huang et al., 2009). We designed a CRISPR/Cas9 construct to target *DEP1* in a similar region of exon 5 (bases 602 to 624 from ATG in cDNA), which was expected to produce effects like those described above in the mutant plants (**Figure 2A**). After introducing the construct into rice embryogenic calli by *Agrobacterium*-mediated transformation, we obtained more than 100 independent regenerated transgenic lines. Next, we examined the mutation efficiency by both PvuII and CEL I digestion of the PCR products amplified from the target site (the target region has a PvuII site) (**Figure 2B**). We found 27 mutant lines among 40 transformants (a mutation rate of 67.5%, **Table 2**). Sequencing of the mutated region confirmed this result (**Figure 2C**). The mutant lines were grown in the field and the phenotypes of homozygous T2 mutants (with no off-targeting events) were investigated. We found that two frame-shift mutations, *dep1*-2 (2-base deletion) and *dep1-4* (4-base deletion), in Zhonghua 11 resulted in decreased plant height and short panicles, but an increased number of flowers per panicle (**Figures 2D–H**). Wild-type panicles have an average length of 17.2 cm, while the lengths of the panicles from mutant lines ranged from 12.8 to 14 cm. On average, 104 flowers were present on the main panicle in wild-type plants, while more than 150 flowers per panicle were scored in the mutant plants (**Figure 2G**). We also found that the grain size (23.1 mg per grain on average) was significantly smaller than that in wild-type plants (25.5 mg per grain on average). As a control, the *dep1-3* mutant, which has a 3-base deletion resulting in a single amino acid deletion, showed a similar phenotype to that of wild-type. These results suggest that mutation of *DEP1* can cause dense panicles in Zhonghua 11, a phenotype consistent with a previous finding in Shennong 265 (Huang et al., 2009). However, the grain size and plant height characteristics of the mutant lines in our study showed some differences from those in the work of Huang et al. (2009).

### Generation of GS3 Mutations and Phenotype of the Mutants

**FIGURE 3:**
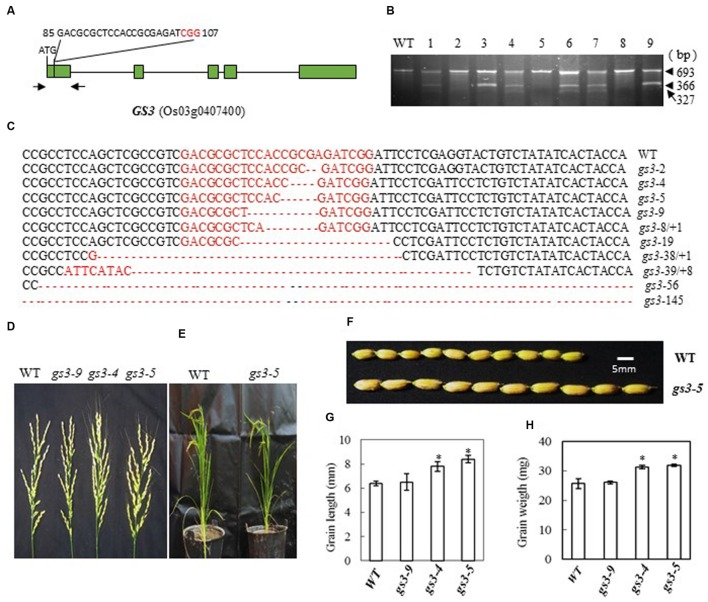
**CRISPR/Cas9-induced *gs3* mutant plants and phenotype analysis. (A)** Schematic map of the genomic region of *GS3* and the sgRNA target site; arrows show the positions of PCR primers used for mutation detection; The PAM motif (NGG) is shown in red; **(B)** Gel electrophoresis of PCR products amplified from the mutated region digested with CEL I; WT and 1–9 are DNA samples from wild type and different transgenic plants. Arrows show the expected band sizes after CEL I digestion. **(C)** Sequence alignment of the sgRNA target region showing altered bases in different mutant lines; **(D)** Representative pictures showing the morphology of the main panicle; **(E)** Phenotype of the mutant plants grown in a greenhouse; **(F)** Comparison of the grain size of mutant *gs3-5* plants with that of WT plants; statistics for the average grain length **(G)** and average grain weight **(H)** of representative mutant plants. Data were collected from 10 to 15 plants per mutant line. * indicates a significant difference (*P* < 0.05) in comparison to WT counterparts.

In Minghui 63, a mutant *GS3* allele with a C→A substitution 165 bp downstream from the predicted translation start site (ATG) causes premature termination of the predicted protein and results in a long-grain phenotype (Mao et al., 2010). The CRISPR/Cas9 construct that we designed to target *GS3* contained single guide RNAs (bases 85 to 107 from ATG in cDNA) with the aim of creating defined deletions in this region (**Figure 3A**). After introducing the construct into rice embryogenic calli by *Agrobacterium*-mediated transformation, we obtained 56 independent regenerated transgenic lines. We examined the mutation efficiency by CEL I digestion of the PCR products amplified from the target site (**Figure 3B**). We found 23 mutants out of 40 transgenic plants, a mutation rate of 57.5%, **Table 2**. The mutations were confirmed by sequencing the targeted region (**Figure 3C**). The mutant lines were grown in the field and the phenotypes of homozygous T2 mutants (with no off-targeting events) were investigated. We found that frame-shift mutants of *GS3* in Zhonghua 11, *gs3-4* (4-base deletion) and *gs3-5* (5-base deletion), showed larger grain size and had long awns on the husks (**Figures 3D–F**). The grain length in the mutants was significantly increased to more than 8 mm compared with the wild-type average of 6.4 mm, and consequently the weight of the grain was increased to more than 31 mg on average, in contrast with 25.7 mg in wild-type (**Figures 3G,H**). The number of flowers per panicle showed no significant difference from the wild-type. As a control, the mutant *gs3-9*, with a 9-base deletion which results in the deletion of three amino acids, showed a similar phenotype to that of wild-type plants. These results suggest that mutation of *GS3* in Zhonghua 11 can increase grain size, a phenotype that was also found in Minghui 63 (Mao et al., 2010). However, the long awn of the grain and the elongated panicle of the mutant indicate that the same gene might function differently in a different cultivar.

### Generation of IPA1 Mutations and Phenotype of the Mutants

**FIGURE 4:**
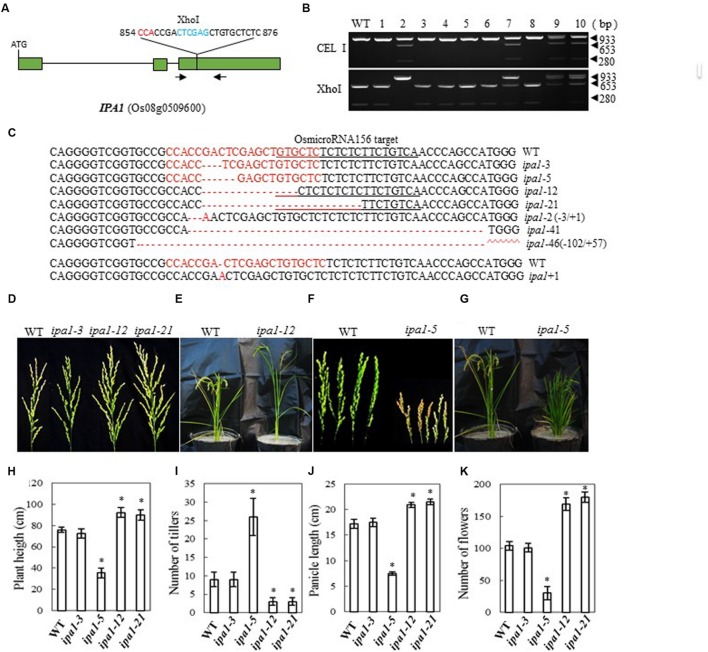
**CRISPR/Cas9-induced *ipa1* mutant plants and phenotype analysis. (A)** Schematic map of the genomic region of *IPA1* and the sgRNA target site; arrows show the positions of PCR primers used for mutation detection; The PAM motif (NGG) is shown in red; Restriction site is shown in blue; **(B)** Gel electrophoresis of PCR products amplified from the mutated region digested with CEL I (upper panel) or XhoI (lower panel); WT and 1–10 are DNA samples from wild type and different transgenic plants. Arrows show the expected band sizes after CEL I or XhoI digestion; **(C)** Sequence alignment of the sgRNA target region showing altered bases in different mutant lines; **(D)** Representative pictures showing the morphology of the main panicle of wild type plants and mutants with the IPA phenotype; **(E)** Wild type and mutant plant with IPA phenotype grown in a greenhouse; **(F)** Panicle morphology of wild type plants and *ipa1-5* mutant plants with a frame-shift in the coding region; **(G)** Phenotype of wild type, and *ipa1-5* mutant plants with a frame-shift in the coding region, in the greenhouse; statistics for plant height **(H)**, number of tillers **(I)**, panicle length **(J)**, and number of flowers **(K)** of representative mutant plants. Data were collected from 10 to 15 plants per mutant line. * indicates a significant difference (*P* < 0.05) in comparison to WT controls.

In Shaoniejing, a C→A substitution at 874 bp in the third exon of *OsSPL14* leads to an amino acid change from leucine to isoleucine and interrupts the cleavage of OsSPL14 transcripts by OsmiR156, generating an ‘ideal’ rice plant with reduced tiller number, increased lodging resistance and enhanced grain yield (Jiao et al., 2010; Miura et al., 2010). The CRISPR/Cas9 construct that we designed to target *IPA1* is close to the region containing the OsmiR156 target site (bases 854 to 876 from the ATG codon in the cDNA, **Figure 4A**). After introducing the construct into rice embryogenic calli by *Agrobacterium*-mediated transformation, we obtained 62 independent regenerated transgenic lines. We examined the efficiency of mutation by both CEL I and XhoI digestion of the PCR products amplified from the target site (there is an XhoI site in the target region) (**Figure 4B**). We found 11 mutants among 40 plants checked, a mutation rate of 27.5% (**Table 2**). The mutants were confirmed by sequencing the mutated region and aligning it with the OsmiR156 target site (**Figure 4C**). The mutant lines were grown in the field and the phenotypes of homozygous T2 mutants (with no off-target events) were investigated. We found that mutation of *IPA1* in the Zhonghua 11 background resulted in three main classes of phenotype, depending on the nature of the mutation: (1) Where the change neither induces a frame shift in the protein coding region nor influences the OsmiR156 target site, the phenotype is wild-type (e.g., *ipa1-3*, which has a 3-base deletion); (2) An IPA phenotype resulted from a deletion of 12 or 21 bases (in *ipa1-12* and *ipa1-21*), which led to amino acid deletions but no frame shift, maintained the activity of the protein and changed the OsmiR156 target site in the IPA1 mRNA (**Figures 4D,E**). The IPA phenotype in the Zhonghua 11 background generally has 2~4 tillers, which is much fewer than the 7–10 tillers typical of wild-type plants, but the plant height, flower number and panicle length are all increased compared with those of wild-type (**Figures 4D–K**). (3) Where base deletions cause a frame shift in the protein (as in *ipa1-5)* which may completely inactivate the protein, the mutant plants have a dwarf phenotype with an increased number of tillers (**Figures 4F,G**). Thus the *IPA1* gene phenotype can be manipulated in the Zhonghua 11 background, but the resulting phenotype varies according to the type of mutation induced.

### Characteristics of Mutations in Rice Induced Using CRISPR/Cas9

Although CRISPR/Cas9 has been successfully used for targeted mutagenesis in rice, its mutation efficiency and the types of mutation it produces still need further investigation. After transformation of our constructs into rice callus and regeneration of transgenic plants, many Cas9-positive T0 plants were identified for each of the targets, and these plants were analyzed to detect mutations in the targeted sequence regions (**Table 2**). For each construct, 40 regenerated plants were examined for the presence of the mutation and used for sequencing. The mutation rate varied widely, from 27.5 to 67.5%, depending on the target gene. On average a mutation rate of 48.75% was obtained in T0 plants. In the T0 generation, homozygous mutations were obtained at frequencies of 12.5% for *Gn1a*, 40% for *DEP1*, 27.5% for *GS3*, and 7.5% for *IPA1*. Bi-allelic mutations were found at frequencies of 5.0% for *Gn1a*, 12.5% for *DEP1*, 7.5% for *GS3*, and 5.0% for *IPA1*.

**FIGURE 5:**
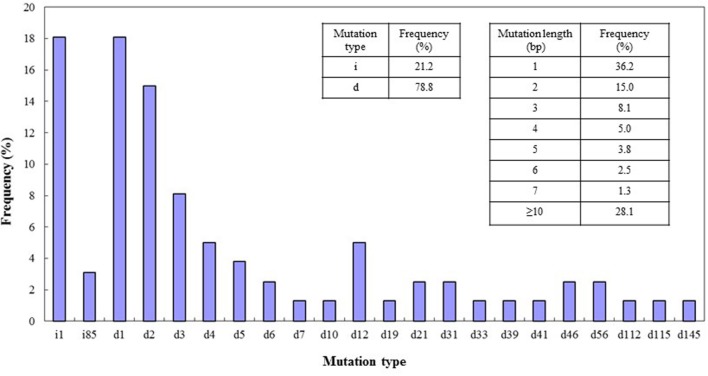
**CRISPR/Cas9-induced mutation types and frequencies.** Left insert, frequencies of mutations of the deletion (d) and insertion (i) types. Right insert, frequencies of different lengths of mutation. On *x*-axis: d#, # of base pairs (bp) deleted from target site; i#, # of bp inserted at target site. Data were collected from lines mutated in all four of the target genes.

The types of mutation and their frequencies were analyzed for the four target sites. Both deletions and insertions of different bases were found in these target sites. Among the mutations, deletions occurred more frequently (78.8%) than insertions (21.2%), and most of the mutations were deletions of 1 or 2 bp or insertions of 1 bp. Mutations or deletions of between 3 and 7 bases were present at frequencies ranging from 1.3 to 8.1%. The percentage of mutations of lengths greater than 10 bp was 28.1% (**Figure 5**). We also detected insertion of a large fragment (85 bp) in the *DEP1* mutation site (**Figure 2C**) and large deletions of 112 and 115 bp from the *Gn1a* sites (**Figure 1C**). Cas9 cleaves double-stranded DNA at a position three base pairs upstream of the protospacer adjacent motif (PAM) sequence (Jinek et al., 2012), and the 1-bp deletions and 1-bp insertions occurred just upstream of this DSB position, at the 4th base from the PAM site.

### Off-target Events

Previous studies showed that off-targets were mutated by CRISPR/Cas9 in a number of different species (Zhang et al., 2014; Zhou et al., 2014; Ma et al., 2015). The major consideration is the homology between the sgRNA and the off-target sites, as well as the length of the sgRNA. To find out whether off-targeting had taken place in our experiments, we searched the rice genome for putative off-target sites which showed high homology with the four sgRNAs, and looked for off-target events in two sites for each sgRNA (**Table 3**). By screening the regenerated plants, we found that three of the four sgRNAs had produced off-target events. For *Gn1a* sgRNA, one of its off-target sites showed high homology with the *Gn1a* site, with only two base differences (12 and 19 base pairs away from the PAM), and this site showed an off-target mutation frequency of 67%. For *IPA1* sgRNA, an off-target site that showed high homology with the *IPA1* site, having only one base difference (8 base pairs away from the PAM), exhibited a mutation frequency of 47.5%. In contrast, in the case of *DEP1* sgRNA, one of its off-target sites, which had a relatively low homology with the *DEP1* site (6 bases were different), showed an off-target mutation frequency of only 2.5%. Sequencing the off-target sites revealed that all the mutations induced in them consisted of single base insertions. These results indicate that there must be a difference of at least two bases between the sgRNA and the target sequence if off-targeting events are to be avoided.

**Table 3:** Mutations detected in putative CRISPR/Cas9 off-target sites.

Target	Putative off-target locus	Sequence of the putative off-target site	No. of mismatching bases	No. of plants sequenced	No. of plants with mutations	Mutation rate (%)
*Gn1a*	Chr5:18581209-18581231	CACCGTCATCCGCGCCGACGAGG	4	40	0	0
	Chr10:3084296-3084316	ACCGCTCCTCCGCGCCGACGAGG	2	40	27	67.5
*DEP1*	Chr1:39674205-39674220	ACAGCTCAAGCAGCTGTACAGGG	6	40	1	2.5
	Chr11:23847196-23847218	TCCCATGAATCAGCTGTACAGGG	5	40	0	0
*GS3*	Chr5:20162701-20162723	GTCGAAGGCCACCGCGAGATCGG	6	40	0	0
	Chr11:19608106-19608128	GATCGACCCCACGGCGAGATCGG	6	40	0	0
*IPA1*	Chr1:30959378-30959400	CGGCGACGTGCTCGAGTCGGTGG	8	40	0	0
	Chr:9:8896084 -8896099	GAGAGCACAGCTGGAGTCGGTGG	1	40	19	47.5

*The PAM motif (NGG) is shown in orange; mismatching bases are shown in red.*

## Discussion

CRISPR/Cas9 has been applied as an efficient targeted mutation method in several different species (Cong et al., 2013; Feng et al., 2013; Jiang et al., 2013; Mali et al., 2013; Nekrasov et al., 2013; Shan et al., 2013). Here, we show that this method can be successfully applied for the mutation of four yield-related genes in a single cultivar, where it produced similar phenotypes to those of previously reported mutants in different cultivars. This finding will facilitate the pyramiding of useful genes into a single cultivar for either breeding purposes or dissection of gene regulatory networks. Transgenes can be segregated out through selfing and thus non-transgenic cultivars can be obtained. The introduction of combinations of these mutations into single cultivar could improve the rice yield. For example, a variety with IPA1 and GS3 mutation might improve rice yield by increasing the effective tillers and the size of the grain.

### Yield-related Genes Offering the Potential for Manipulating Yield-related Traits in Different Rice Cultivars

Mutagenesis is a powerful method with which to study genes and gene networks related to plant development. Generally, natural mutations are found in different cultivars with different genomic backgrounds. High-yield mutants have been intensively studied, leading to the characterization of many genes related to high-yield traits (Ikeda et al., 2013). However, whether these mutant genes can function in different cultivars and thus be widely used in rice breeding needs further investigation. In this work, four yield-related genes (*Gn1a*, *DEP1*, *GS3*, and *IPA1*) were mutated in the Zhonghua 11 background using the CRISPR/Cas9 method. The phenotypes of the mutants suggested that the four genes have similar functions in different genomic backgrounds, though there are some differences.

*Gn1a* encodes a cytokinin oxidase, and mutations in the promoter and coding regions of the gene were previously shown to result in an increased number of grains and large panicles (Ashikari et al., 2005). Here we introduced a frame-shift by mutating the gene, and obtained a similar phenotype in the Zhonghua 11 background to that observed by Ashikari et al. (2005), with large panicles and increased numbers of flowers (**Figures 1D,G,H**). The average number of flowers per panicle in the two mutants was, at 184 and 199, almost twice that in wild type. Map based cloning of *Gn1a* was previously carried out in an *indica* rice variety, Habataki, which has about 306 flowers per panicle; when crossed with a *japonica* cultivar (164 flowers per panicle), the resulting hybrids produced about 405 flowers per panicle. In this case, both *Gn1a* and hybrid vigor may have contributed to the increased flower number in the hybrids (Ashikari et al., 2005). We also observed a minor reduction of tillering in the mutants, and tillering occurred after heading in the basal part and in the uppermost nodes. A *DEP1* mutant in the Shennong 265 background displays a dense erect panicle, and increases rice yield by 15–20% (Huang et al., 2009). Recent results showed that DEP1 interacted with the subunits Gα (RGA1) and Gβ (RGB1) of the rice heterotrimeric G-protein, and reduced RGA1 activity or enhanced RGB1 activity inhibits nitrogen responses (Sun et al., 2014). Mutation of *DEP1* with CRISPR in Zhonghua 11 produced a similar phenotype, with short, dense, erect panicles (**Figure 2D**), and also increased the number of flowers per main panicle (**Figure 2G**). However, the size of the grain and the plant height were reduced (**Figures 2E,F**), which suggests that this gene influences plant growth and seed development, in addition to panicle architecture. Previous studies by different groups showed that *dep1* confers an erect panicle character in *japonica* rice, and also leads to phenotypes such as reduced plant height, grain size, and tillering. Controversial effects on yield were observed when *dep1* was introduced into different cultivars, which suggests that the erect panicle allele should be used together with other favorable genes in order to breed for high yield (Huang et al., 2009; Taguchi-Shiobara et al., 2011; Yi et al., 2011). For *GS3*, we obtained a mutant phenotype with large grain size (**Figures 3D,F**), which is similar to the original *GS3* phenotype in the *indica* variety Minghui 63 (Mao et al., 2010). The average grain length in Minghui 63 is 10.4 mm, whereas the grain length is only 7.4 mm in another *indica* variety, Chuan 7, which has functional GS3 (Fan et al., 2006). Additionally, we observed long awns on the grains of the mutant (**Figure 3D**). For *IPA1*, we produced different phenotypes depending on whether we changed the miR156 target site in the IPA1 coding sequence or mutated the protein (**Figures 4D–G**). Two mutants with deletions of 12 and 21 bp in the IPA1 coding sequence showed a phenotype with less tillering, more grains and a higher frequency of seed set (**Figures 4D,H–K**).

### Targeted Mutation of the miR156 Site in IPA1 Produced Multiple Phenotypes

MicroRNAs are important regulators of gene expression and involved in many aspects of plant growth and development (Voinnet, 2009; Rogers and Chen, 2013). miR156, one of the most conserved and highly expressed microRNAs in plants, targets *SQUAMOSA-promoter binding-like* (*SPL*) transcription factor genes (Xie et al., 2006, 2012). Overexpression of *miR156* in plants results in dramatic morphological alterations, e.g., dwarf or bushy architecture, reduction in seed or tuber yields, less nodulation, and delayed flowering, suggesting that miR156 has multiple regulatory roles in plant development (Xie et al., 2006; Chuck et al., 2007; Hultquist and Dorweiler, 2008; Wang et al., 2009; Wei et al., 2010; Bhogale et al., 2014; Stief et al., 2014; Wang Y. et al., 2015).

The *IDEAL PLANT ARCHITECTURE1* (*IPA1*) gene is the most extensively studied SPL (it is also known as *OsSPL14*) controlling tillering in rice (Jiao et al., 2010; Miura et al., 2010). In the japonica line Shaoniejing (SNJ), one point mutation in the recognition site for miR156 perturbs *IPA1* transcriptional cleavage and translational repression, leading to several traits including a decrease in tiller number, and increased plant height and panicle branching (Jiao et al., 2010). The *WEALTHY FARMER’S PANICLE* (*wfp*) was found to be an epigenetic allele of *IPA1* (Miura et al., 2010). The mutant phenotype described above indicates that the *ipa1* phenotype in rice may be achieved by directly knocking a pre-designed mutated miR156 recognition site into *OsSPL14*, rather than through time-consuming back-crossing from an *ipa1* plant. Here we mutated the miR156 target site in *IPA1* using CRISPR/Cas9, and we found a similar phenotype to that of *ipa1* plants; sequencing the mutant lines revealed that they contain deletions of 12 and 21 base pairs, which disrupt the miR156 sites (**Figure 4C**), so that the *IPA1* transcripts can survive attack by microRNA156, while deletion of amino acids in this region does not influence the activity of the IPA1 protein. Thus, CRISPR/Cas9 is a potentially powerful method with which to mutate microRNAs and their targets in order to elucidate microRNA regulatory networks. We also obtained many *IPA1* mutants with frameshifts, which abolished IPA1 activity; these plants were dwarf and had more tillers, resembling the phenotype of plants over-expressing miR156 (**Figure 4G**). We are now carrying out experiments to mutate miRNA156s and their target genes in rice.

### CRISPR/Cas9-Mediated Mutation Types and Off-target Events

Although CRISPR/Cas9 has been applied in many species, its efficiency and the types of mutation it produces vary (Cong et al., 2013; Jiang et al., 2013; Mali et al., 2013; Nekrasov et al., 2013; Zhang et al., 2014; Zhou et al., 2014; Ma et al., 2015). Based on our results with the four target sites and the editing events observed, we concluded that this system edits at high efficiency in rice. The reason for this high efficiency could be the fact that Cas9 is codon optimized and expressed at a high level (Ma et al., 2015). We obtained different types of mutation from those reported in some previous work (Zhang et al., 2014; Zhou et al., 2014; Ma et al., 2015). In a study by Zhang et al. (2014), mutations with a single base insertion constituted most of the events, whereas our results showed that deletion events are more frequent than insertions (78.8% vs 21.2%) and 28.1% of mutants had insertions or deletions of more than 10 bp (**Figure 5**). The reasons for these inconsistencies could be different levels of Cas9 activity or expression, sgRNA expression level, and/or the base composition of the target sequence (Zhang et al., 2014; Zhou et al., 2014; Ma et al., 2015). Interestingly, the off-targets in our experiments all showed insertions of 1 base pair, which indicates that the pairing of sgRNA with the target sequence may also influence the mutation type.

As our research aimed to compare the functions of known genes in a different genomic background, we designed the sgRNA target sites on the basis of the mutation sites described in previous studies on the same genes. To investigate whether off-targeting had taken place in our transgenic plants, we searched the rice genome for sequences highly similar to the sgRNA target sites. We detected off-target events in three of the four sgRNAs. The highest off-target rate, 67.5%, occurred in the case of *Gn1a*, where there was a mismatch of two bases between the sgRNA and its target sequence. A 47.5% off-targeting rate was observed for *IPA1*, with a single mismatched base between the sgRNA and its target sequence. A 2.5% off-target rate was found for *DEP1* sgRNA, where there were six mismatched bases between the sgRNA and its target sequence (**Table 3**). Generally the off-target events are related to the sequence homology between the sgRNA and the target sequence, as well as the position of the mismatched bases. Previous studies on human cells showed that single and double mismatches can cause high frequencies of off-target events, and off-target sites harbored up to five mismatches were identified as being mutagenized at frequencies comparable to (or higher than) those observed at the intended on-target site (Fu et al., 2013). Off-target events are also found in plants, where they have been exploited in order to mutagenize multiple sites with a single sgRNA (Endo et al., 2015). Our results suggest that more than two base mismatches between a sgRNA and an off-target site are needed in order to avoid off-target events when CRISPR/Cas9 is used in rice. As CRISPR technology develops, new strategies such as the use of CRISPR-Cas9 nickase and controlling the expression of *Cas9* with specific promoters should greatly improve its efficiency (Shen et al., 2014; Yan et al., 2015). Moreover, off-target mutations in plants can be segregated out by traditional breeding.

### CRISPR/Cas9-Mediated Editing of MicroRNA Sites

MicroRNA target sites are generally 21 bp in length and it is difficult to obtain desired mutations in them for specific purposes. For example, the natural mutation in *IPA1* has only a single base alteration, which results in an IPA phenotype; the single base change alters the micro156 target site but the function of the IPA1 protein is still retained. To obtain a similar mutation by existing methods is difficult, and other methods such as antisense or RNAi suppression of miR156 may have additional effects, since miR156 may have multiple targets. In addition, suppression of gene expression by antisensing or RNAi may be incomplete. Our work has shown that a CRISPR/Cas9 strategy may be ideally suited to overcoming the limitations of these other approaches. In our experiments, we obtained different phenotypes by mutating the miR156 target site in *IPA1*. Targeted deletion of either 12 or 21 bp from the *IPA1* transcript region produced the IPA phenotype, by disrupting the miR156 target site while keeping the remaining codons in frame and thus maintaining protein activity. Other mutations, those that introduced frame-shifts, resulted in a contrasting phenotype, which was dwarf and had more tillers. Our work on targeted modification of miR156 target sites has shown that CRISPR/Cas9 is a powerful method for manipulating microRNA or its targets.

## Conclusion

We have used a CRISPR/Cas9 system to edit four yield-related genes in Zhonghua 11 rice, and obtained phenotypes similar to those of previously reported mutants (Ashikari et al., 2005; Jiao et al., 2010; Mao et al., 2010; Miura et al., 2010). This finding offers the prospect of efficiently reassessing the roles of yield-related genes in different rice cultivar backgrounds, and it may be possible to directly combine different types of interesting high yield traits in the same genetic background without necessitating the time-consuming production of nearly isogenic lines. Consequently, it may become feasible to pyramid useful genes into a single cultivar for breeding purposes (Ainley et al., 2013). Furthermore, the suitability of this method for manipulating microRNA target sites makes it an effective approach for the dissection of microRNA function.

## Author Contributions

HL designed and carried out the project; GW directed the study; ML, PW, MF, and XP carried out the transgenic plant generation and analysis and evaluated the agronomic trait data; XL and ZZ performed the mutation analysis; QL and WL assisted in the phenotype investigation. ML and HL wrote the manuscript. All authors read and approved the final manuscript.

## Conflict of Interest Statement

The authors declare that the research was conducted in the absence of any commercial or financial relationships that could be construed as a potential conflict of interest.
