# Supplementary material for: Reassessment of the Four Yield-related Genes Gn1a, DEP1, GS3, and IPA1 in Rice Using a CRISPR/Cas9 System
Source: Front Plant Sci. 2016 Mar 30;7:377. doi: 10.3389/fpls.2016.00377 (PMC4811884; doi:10.3389/fpls.2016.00377)
Supplement: Supplementary file 1 [file Presentation_1.PDF]

a. Plasmid map of pYLCRISPR/Cas9(I)

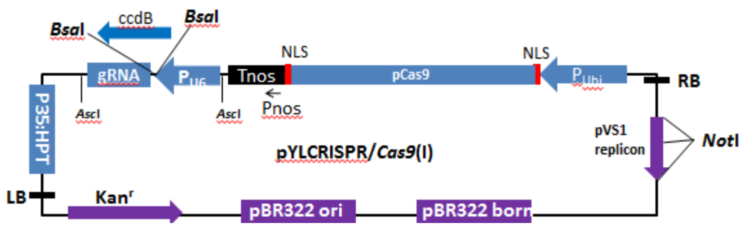

b. Primers for amplification of CRISPR/Cas9 target region

DEP1-Fw	GCCTCTAGTCCCTTTGTATG
DEP1-Re	GAGCAGGAGAAGCAGTCC
Gn1a-Fw	TATAGGCCACCTTGTCCCTTCT
Gn1a-Re	CGACGGTGAGGTGGAGGTAG
GS3-Fw	AAGAAAAGAGCGGCCATCCA
GS3-Re	TGGCTGAGAAGAGAGGAGCA
IPA1-Fw	AAGAGCATCGCAGGTTCA
IPA1-Re	GTCCTCGACAATACAGCATTAA

c. Oligonucleotides for target sequence of sgRNA

DEP1C-Fw	GCCGATCTTGAAGCAGCTGTACA
DEP1C-Re	AAACTGTACAGCTGCTTCAAGAT
Gn1aC-Fw	GCCGCCGCTCATCCGCGCCGACG
Gn1aC-Re	AAACCGGCGCGGATGAGCGGCGG
Gs3C-Fw	GCCGACGCGCTCCACCGCGAGAT
Gs3C-Re	AAACATCTCGCGGTGGAGCGCGT
IPA1C-Fw	GCCGAGAGCACAGCTCGAGTCGG
IPA1C-Re	AAACCCGACTCGAGCTGTGCTCT

d. Primers for amplification of off-target fragments

Off-target sgRNA sequences	Primers for amplification of off-target fragments
ACAGCTCAAGCAGCTGTACAGGG	DEP1-01g-16bp-Fw GCGCAACACAACACCTACA<br>DEP1-01g-16bp-Re GTGATAGCGCCAGCAAAACC
TCCCATGAATCAGCTGTACAGGG	DEP1-11g-18-1-Fw: AGGAGCCAGACTACCAAGGG<br>DEP1-11g-18-1-Re: GATGAGGACCGAATGCGATG
CACCGTCATCCGCGCCGACGAGG	Gn1a-04g-Fw GAGATTCCATTTGCCTTTACAC<br>Gn1a-04g-Re GATGCACCTGATGACCTTCC
ACCGCTCCTCCGCGCCGACGAGG	Gn1a-10g-Fw: CGACACAGCCGTAATGAGGA<br>Gn1a-10g-Re: ACAAGTGAATCATCCCCAACA
GTCGAAGGCCACCGCGAGATCGG	Gs3-05g-Fw TTGAGGTAGAGGCGGTACTTCTGG<br>Gs3-05g-Re CTTCTTTCTTCCTTCTCCGATGC
GATCGACCCCACGGCGAGATCGG	Gs3-11g-FW2: AGAATGAGTATGCAGGTGAT<br>Gs3-11g-RE2: GGAACGCTGGAAGGTTAGGA
CGGCGACGTGCTCGAGTCGGTGG	IPA1-01g-Fw GACGACTCTGCTCTTCGCTT<br>IPA1-01g-Re CGGGTGAGTCGTGTTGAAGT
GAGAGCACAGCTGGAGTCGGTGG	IPA1-09g-Fw: CCGACACCTGGACCTCTTTC<br>IPA1-09g-Re: ACTAGGCTGCCCAATGTTCC
